# Hydrogen Sulfide Alleviates Schizophrenia‐Like Behavior Through Regulating Apoptosis by S‐Sulfhydrylation Modification

**DOI:** 10.1111/cns.70278

**Published:** 2025-02-18

**Authors:** Xinzhe Du, Wei Hu, Meiqi Liu, Jinzhi Lv, Yao Gao, Xiao Wang, Wentao Zhao, Junxia Li, Xinrong Li, Xiaohua Cao, Zhifen Liu, Yong Xu, Sha Liu

**Affiliations:** ^1^ Department of Psychiatry First Hospital of Shanxi Medical University Taiyuan Shanxi China; ^2^ Shanxi Key Laboratory of Artificial Intelligence Assisted Diagnosis and Treatment for Mental Disorder First Hospital of Shanxi Medical University Taiyuan Shanxi China; ^3^ Basic Medical College Shanxi Medical University Taiyuan Shanxi China; ^4^ Obstetrics and Gynecology Department The Second Hospital of Jilin University Changchun Jilin China; ^5^ The Eighth Affiliated Hospital Sun yat‐sen University Guangdong China

**Keywords:** Apoptosis, Dizocilpine, Hydrogen Sulfide, Schizophrenia, S‐sulfhydrylation

## Abstract

**Background:**

We initiated an exploration of the relationship between hydrogen sulfide (H_2_S) and Schizophrenia (SZ) as well as its mechanism at the three levels of population study, cellular investigation, and animal model.

**Materials and Methods:**

Clinical data and peripheral blood samples from 78 patients with SZ and 83 healthy controls (HC) were collected for the detection of H_2_S levels (ChiCTR (Chinese Clinical Trial Registry) 900026776). MK801 (Dizocilpine) was used to establish SZ models in cells and rats, with sodium hydrosulfide (NaHS) serving as an exogenous H_2_S donor. H_2_S levels in plasma and hippocampal tissue of rats were measured using Enzyme Linked Immunosorbent Assay (ELISA). Terminal dUTP Nick End Labeling (TUNEL) staining was employed to detect apoptosis, enzyme activity was determined to assess apoptotic protease activity, neuron damage was identified by Nissl staining, and the protein S‐sulfhydrylation test was utilized to evaluate alterations in apoptosis‐associated protein S‐sulfhydrylation.

**Results:**

H_2_S content significantly decreased in the plasma of SZ patients and in the plasma and hippocampal tissue of SZ model rats. NaHS pretreatment reduced MK801‐induced apoptosis in SH‐SY5Y cells. SZ model rats exhibited increased behavioral abnormalities, hippocampal apoptosis, and reduced S‐sulfhydrylation of an apoptosis‐related protein, both restored after NaHS pretreatment.

**Conclusions:**

H_2_S content is significantly reduced in SZ, and supplementation of H_2_S can alleviate SZ‐like behavior by inducing S‐sulfhydration of apoptotic proteins.

## Graphical abstract

We explored the regulatory impacts and mechanisms of H_2_S on SZ at multiple levels in human, cellular, and animal models. Our findings indicated that H_2_S levels were significantly decreased in SZ patients and SZ model rats. Exogenous H_2_S supplementation might alleviate schizophrenia‐like behaviors by inhibiting apoptosis through S‐sulfhydrylation modification. The graphic abstract is drawn By Figdraw.

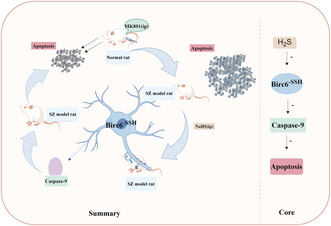

## 1 Introduction

Schizophrenia (SZ) is a disorder primarily distinguished by positive symptoms, negative symptoms, and cognitive impairment [1, 2]. The lifetime prevalence is estimated to be approximately 1% [3, 4]. The social function and quality of life of patients with SZ are severely impaired. The persistence of SZ brings a heavy burden to individuals, families, and society [5]. SZ is a complex disorder influenced by both genetic and environmental factors [6]. Several theories have been suggested to explain its pathogenesis [7]. However, a comprehensive understanding of the mechanisms underlying SZ remains elusive. A lack of reliable diagnostic and treatment markers leads to unsatisfactory therapeutic outcomes, highlighting the necessity for safer and more effective treatment strategies.

In individuals diagnosed with SZ, there is evidence of dysregulated apoptosis within the neural network of the brain. Apoptosis represents a fundamental physiological mechanism that plays a crucial role in the normal development and function of the brain [8]. Levels of apoptotic proteins have been found to be increased in cadaveric brain tissue [9, 10] and the peripheral blood of patients with SZ [11]. Caspases are a family of cysteine proteases that play a crucial role in apoptosis [12], processes that have been implicated in various neuropsychiatric disorders, including SZ [11]. Abnormalities in neuronal apoptosis signaling pathways during the onset of SZ may cause neuronal damage or degeneration, ultimately leading to cognitive decline in patients [13, 14].

In recent years, hydrogen sulfide (H_2_S) has gained increasing attention as a gaseous signaling molecule due to its natural production in the body and important physiological functions [15, 16]. Accumulated evidence indicates that patients suffering from SZ have a reduced H_2_S level in plasma, inversely related to clinical symptoms [17], while *postmortem* analysis suggests increased H_2_S in brain tissue [18]. It has been hypothesized that disturbances in the endogenous H_2_S level may contribute to the development of SZ symptoms.

H_2_S plays a part in the regulation of several neuropsychiatric conditions, including Alzheimer's disease (AD), Parkinson's disease (PD), and depression. H_2_S exhibits antiapoptotic properties that help slow the progression of these disorders. It has been demonstrated that H_2_S can improve deficits in learning and memory by protecting hippocampal neurons through the reduction of apoptosis [19, 20]. The inhibition of apoptosis by H_2_S may result in a slowing down of AD progression [21]. H_2_S can regulate protein activity and function by modifying cysteine residues through a process called S‐sulfhydrylation, which involves the transformation of the sulfhydryl group from‐SH to‐SSH [22, 23]. The S‐sulfhydrylation modification of H_2_S is intricately linked to various neuropsychiatric disorders and has been employed in the investigation of the progression and evolution of different medical conditions [24]. A deficiency of H_2_S is associated with atypical sulfhydrylation, which has been implicated in cognitive decline and the dementia linked to AD [25]. In addition, H_2_S has been found to influence the progression of PD via S‐sulfhydrylation signaling [26]. Consequently, we hypothesized that H_2_S could ameliorate SZ by modulating S‐sulfhydrylation modification to inhibit apoptosis.

**FIGURE 1:**
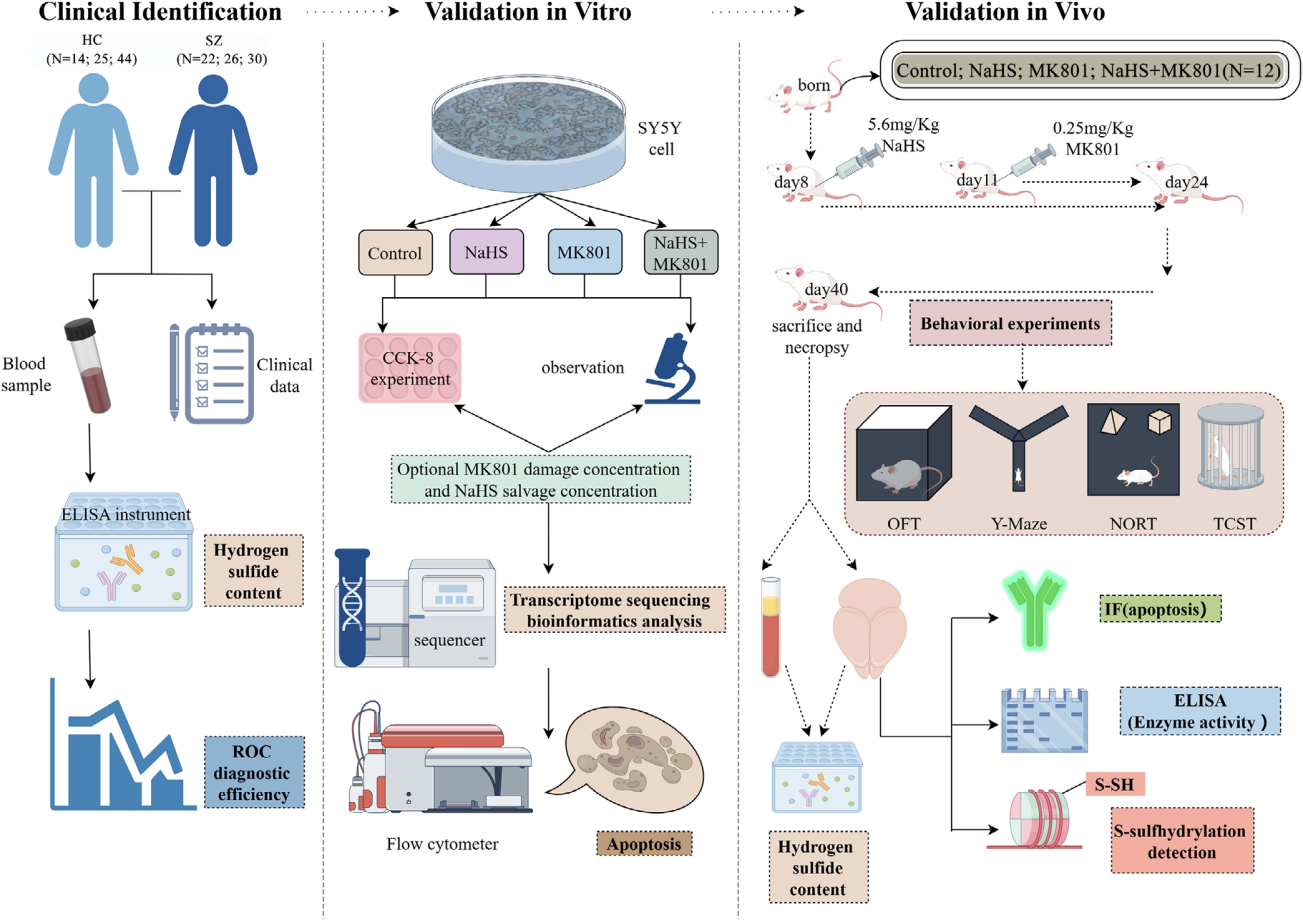
The study flowchart. CCK‐8, Cell Counting Kit‐8; ELISA, Enzyme‐Linked Immunosorbent Assay; HC, healthy controls; IF, Immunofluorescence; Kg, Kilogram; mg, milligram; MK801, Dizocilpine; NaHS, sodium hydrosulfide; NORT, Novel Object Recognition Test; OFT, Open Field Test; ROC, Receiver Operating Characteristic; S‐SH, persulfide group. SY5Y, Human Neuroblastoma Cells; SZ, schizophrenia; TCST, Three‐Chamber Social Test.

In the present study, we first identified changes in the H_2_S level in the peripheral blood of patients with SZ in different population subsets. We confirmed this change in the H_2_S level in a rat model of SZ. Subsequently, the effect of H_2_S on MK801‐induced cell damage was investigated in the SZ cell model. Assays based on immunofluorescence and apoptotic proteinase activity were utilized to investigate the apoptosis status of hippocampal cells in a rat model of SZ. Furthermore, alterations in the levels of S‐sulfhydrylation of hippocampal‐related proteins were investigated. We elucidated the pathogenesis of SZ from the perspective of H_2_S. We also provided a theoretical basis for the clinical application of H_2_S in the treatment of SZ. The technical roadmap of this study is shown in Figure 1 (By Figdraw).

## 2 Materials and Methods

Ethical approval of the population study protocol was granted by the Ethics Committee of First Hospital of Shanxi Medical University (ChiCTR900026776). The cell investigation used SH‐SY5Y (Human Neuroblastoma Cells) from the Shanghai Cell Bank of the Chinese Academy of Sciences (Beijing, China). The animal study protocol was approved (2021‐150) by the Animal Ethics Committee of Shanxi Medical University (Shanxi, China). Sprague–Dawley rats were acquired from SPF (Beijing) BIOTECHNOLOGY Co. Ltd. (Beijing, China). Animals received optimal care in a room at a controlled temperature of 24°C and a relative humidity of 55%.

The detailed description of all materials and methods, including Enzyme Linked Immunosorbent Assay (ELISA), methylene blue method [27, 28], cell recovery and culture, Cell Counting Kit (CCK)8 assay, high‐throughput sequencing of microRNAs (miRNAs) and messenger RNA(Ribonucleic Acid) (mRNA), cytotoxicity assay, behavior assay, Open Field Test (OFT), Y‐Maze Test (YMT), Three‐Chamber Social Test (TCST), Novel Object Recognition Test (NORT), Terminal deoxynucleotidyl transferase deoxyUridine Triphosphate Nick End Labeling (TUNEL) staining, caspase‐9 activity analysis and S‐sulfhydrylation detection can be found in the Supplement information.

The data were analyzed using International Business Machines Corporation (IBM) Statistical Package for the Social Sciences (SPSS) (version 20) and plotted using GraphPad Prism 9. Chi‐square tests were used to compare categorical variables during data analysis. The interaction test was performed using between‐subject effects analysis. The Shapiro‐Wilk test (SW) was first used to assess the normality of the data for continuous variables. The independent samples t‐test was used to assess the difference between the two data groups if the data followed a normal distribution. One‐way Analysis of Variance (ANOVA) was used to compare multiple sets of data. In the homogeneity of variance test, if the variance is homogeneous, the Least Significant Difference (LSD) is used for the post hoc analysis; otherwise, Tamhane's T2 test is used. The Mann–Whitney U test was used to assess differences between groups when the data were not normally distributed. Receiver operating characteristic (ROC) curve analysis served as an adjunct in evaluating efficacy. A significance level of *p* < 0.05 indicated statistical significance.

## 3 Results

### 3.1 Human Study

#### 3.1.1 Plasma H_2_S Content Was Significantly Decreased in Patients With SZ

**FIGURE 2:**
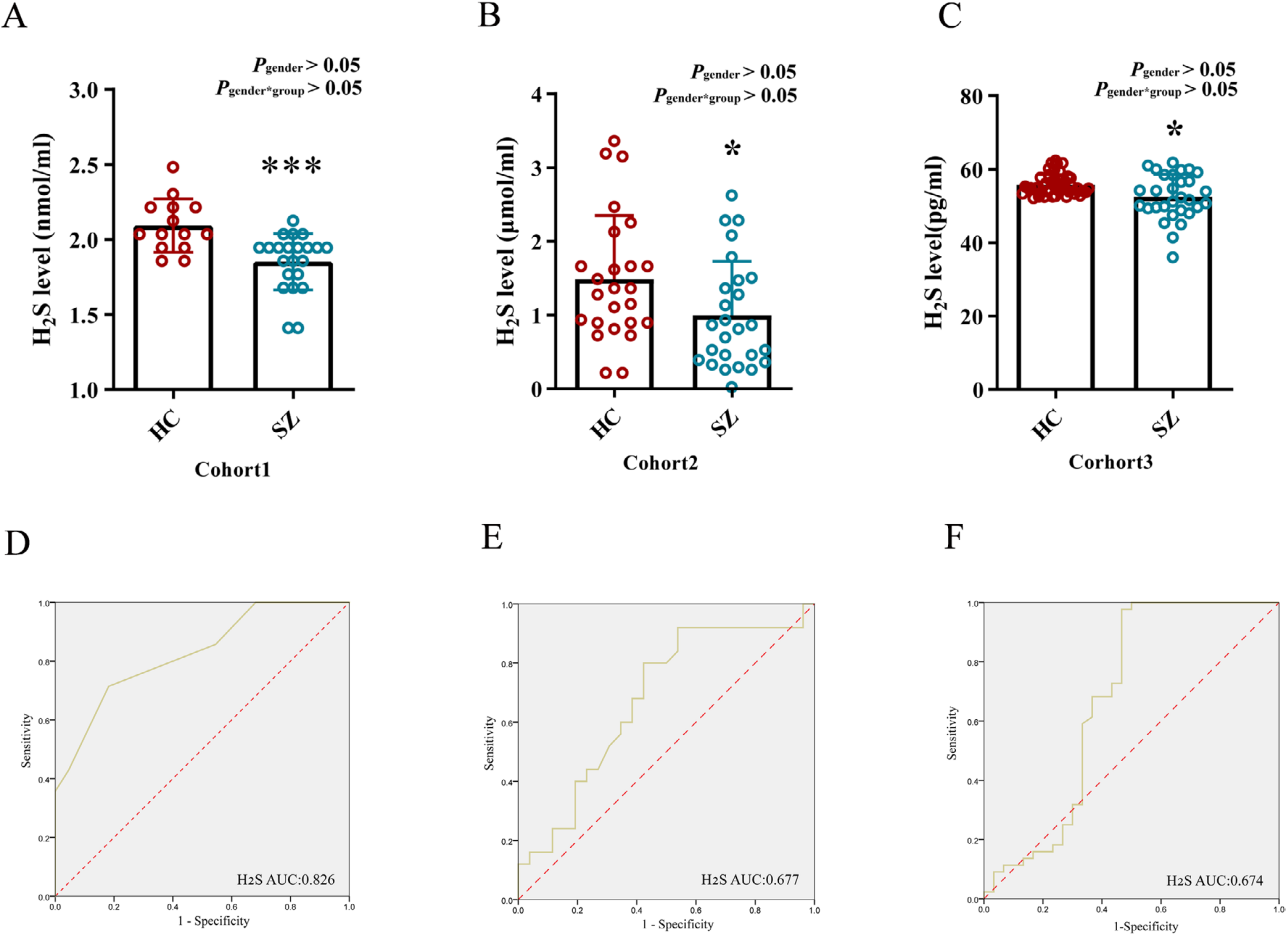
Plasma H_2_S level and ROC curve analysis in three groups of population cohorts. (A–C) Plasma H_2_S levels in SZ and HC. (A) Cohort1: HC (*N* = 14), SZ (*N* = 22). Mann–Whitney U test, ***p < 0.001 vs. HC. (B) Cohort2: HC (*N* = 25), SZ (*N* = 26). Mann–Whitney U test, *p < 0.05 vs. HC. (C) Cohort3: HC (*N* = 44), SZ (*N* = 30). Mann–Whitney U test, *p < 0.05 vs. HC. (D–F) ROC curves of plasma H_2_S content and SZ in three groups of cohorts. AUC, Area Under the Curve; H_2_S, hydrogen sulfide; HC, healthy controls; ROC, Receiver Operating Characteristic; SZ, schizophrenia.

This research utilized the initial two clinical sample groups as a discovery cohort for H_2_S analysis and a third group as an independent validation cohort to verify H_2_S content variations in SZ patients. Chi‐square tests showed no significant differences in sex, age, educational attainment, body mass index (BMI) diastolic blood pressure (DBP) and systolic blood pressure (SBP) between patients and controls. None of the patients or controls were drug or substance abusers. All patients had no prevalence of comorbidity (Table 1). H_2_S level in peripheral blood was measured in 22 patients with SZ and in 14 Healthy Controls (HC) using the methylene‐blue method. The data revealed a notable reduction in the plasma H_2_S level in the patient group (Figure 2A). A subsequent ELISA on 26 patients and 25 controls confirmed these findings (Figure 2B). A further validation cohort of 44 patients and 30 HC also showed a reduced H_2_S level in patients suffering from SZ (Figure 2C). Additionally, the potential impact of gender on H_2_S content were examined. The tests of Between‐Subjects Effects analysis showed that gender did not exert a significant primary effect on the disparity in H_2_S content and there was no interaction between gender and group suggesting that gender was not a major contributing factor to the variation in H_2_S content in this study (Figure 2A–C). The diagnostic efficacy of H_2_S showed an area under the receiver operating characteristic (ROC) curve (AUC) of 0.826, 0.677, and 0.674, sensitivity of 71.4%, 92%, and 97.7%, and specificity of 81.81%, 46.15%, and 53.33% for Cohort1, Cohort2, and Cohort3, respectively (Figure 2D–F). These data suggested H_2_S to be a potential plasma biomarker for the diagnosis of SZ.

**TABLE 1:** Comparison of basic data of SZ and HC in three population cohorts.

	HC(*N* = 83)	SZ(*N* = 78)	Z/χ2/t	*P*
Cohort1	HC(*N* = 14)	SZ(*N* = 22)		
Age (year) M(IQR)	31 (19.5,43.75)	32.5 (21.75,39.75)	0.308^a^	0.760
Education level (year) M(IQR)	12 (9,12)	12 (9,12)	−0.144^a^	0.885
Gender (male/female)	4/10	8/14	0.234^b^	0.629
BMI M(IQR)	23.85 (20.89,27.61)	23.70 (20.53,27.83)	−0.342^c^	0.735
DBP M(IQR)	77.5 (71.25,85)	78 (67.75,81.75)	0.230^c^	0.820
SBP M(IQR)	117 (108.75,127.25)	119 (109.75,127.5)	−0.483^c^	0.632
Substance dependence or abuse (%)	0	0		
Prevalence of comorbidity (%)		0		
PANSS score		72.15 ± 17.74		

Cohort2	HC(*N* = 25)	SZ(*N* = 26)		
Age(year) M(IQR)	26 (23.5,31.5)	26 (22,35.5)	−0.113^a^	0.910
Education level(year) M(IQR)	15 (12,16)	12 (11.25,15.25)	−1.406^a^	0.160
Gender(male/female)	8/17	11/15	0.579^b^	0.447
BMI M(IQR)	22.66 (20.76,25.49)	22.48 (19.51,26.36)	0.016^c^	0.988
DBP M(IQR)	73 (69,81)	80 (73.5,82.5)	−1.862^c^	0.069
SBP M(IQR)	112 (108.5122)	120 (111,124.5)	−1.499^c^	0.140
Substance dependence or abuse (%)	0	0		
Prevalence of comorbidity (%)		0		
PANSS score		84.42 ± 16.21		

Cohort3	HC(*N* = 44)	SZ(*N* = 30)		
Age(year) M(IQR)	17 (15.25,17)	15 (13.75,16.25)	−1.915^a^	0.055
Education level (year) M(IQR)	12 (11.25,12)	12 (9,12)	−0.691^a^	0.489
Gender(male/female)	22/22	9/21	2.931^b^	0.087
BMI M(IQR)	21.77 (18.56,23.06)	20.30 (18,21.58)	1.228^c^	0.224
DBP M(IQR)	71 (65.75,80)	70 (65.75,77)	0.851^c^	0.398
SBP M(IQR)	107.5 (99.75,116)	104.5 (96.5112,25)	0.908^c^	0.367
Substance dependence or abuse (%)	0	0		
Prevalence of comorbidity (%)		0		
PANSS score		91.17 ± 35.24		

**Abbreviations:** HC, healthy controls; SZ, schizophrenia; M, Median; IQR, interquartile range; BMI, Body Mass Index; BP, Blood Pressure; DBP, Diastolic Blood Pressure; SBP, Systolic Blood Pressure; PANSS, Positive and Negative Syndrome Scale.

^a^ Mann–Whitney U test.

^b^ χ2 test.

^c^ T‐test. Z/χ2/t/*P* is a statistical value.

### 3.2 Cell Study

#### 3.2.1 H_2_S Alleviates Damage Induced by MK801 in SH‐SY5Y Cells

**FIGURE 3:**
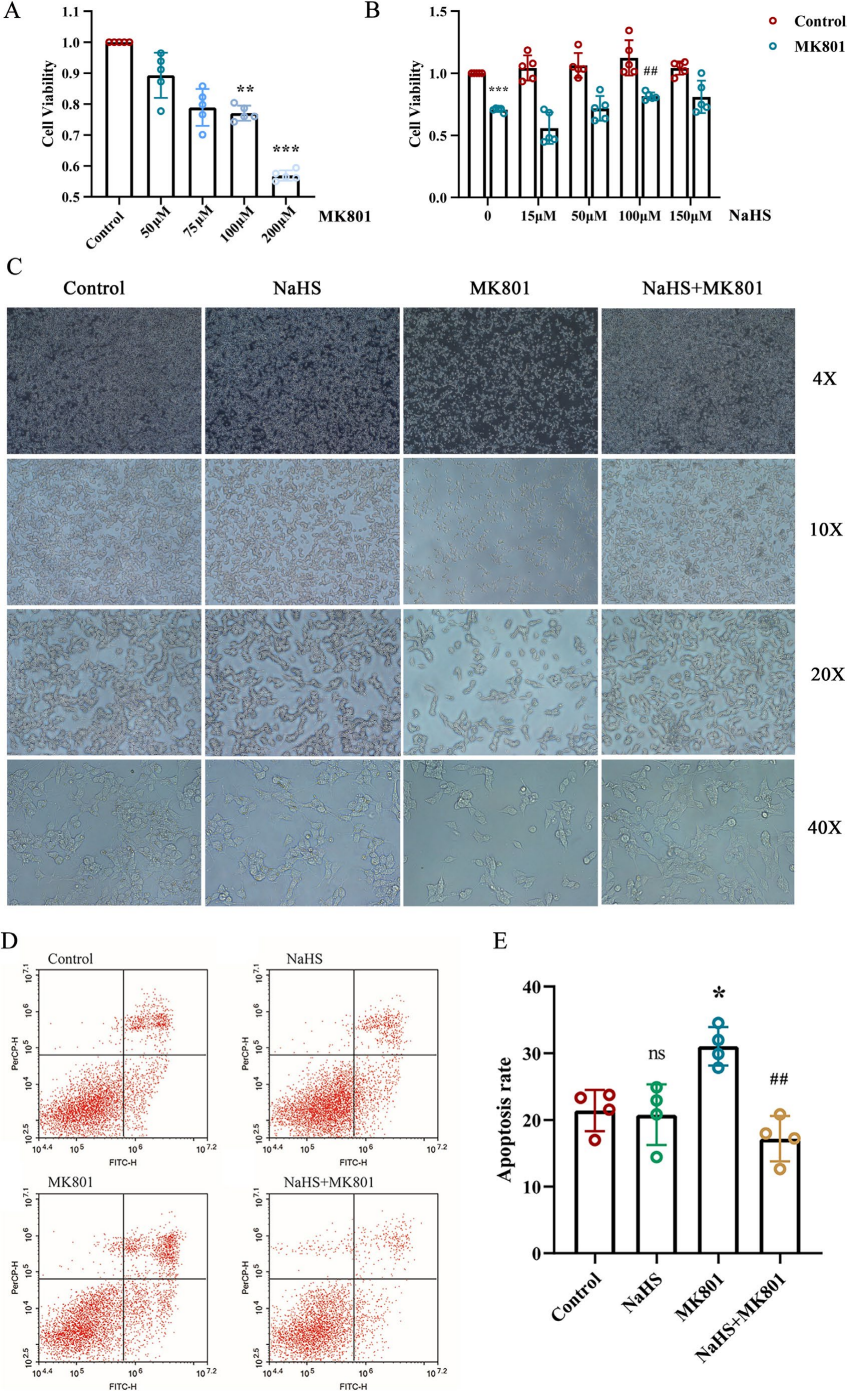
Effect of H_2_S on damage caused by MK801 in SH‐SY5Y Cells. (A) Effects of different concentrations of MK801 on the viability of SH‐SY5Y cells. *N* = 5. One‐way ANOVA followed by Tamhane's T2 test post hoc analysis. *p < 0.05, **p < 0.01, ***p < 0.001 vs. Control. (B) Effect of NaHS on MK801‐induced cell viability injury. *N* = 5. Mann–Whitney U test, ***p < 0.001 vs. Control: ^##^ *p* < 0.01 vs. MK801. (C) NaHS alleviates cell morphological damage induced by MK801 (4×, 10×, 20×, and 40× field of view). (D) Representative apoptotic images of different groups. (E) Statistical results of flow cell apoptosis. *N* = 4. One‐way ANOVA followed by Least Significant Difference (LSD) post hoc analysis, *p < 0.05, ns (no significance) vs. Control, ^##^ *p* < 0.01 vs. MK801. ANOVA, Analysis of Variance; MK801, Dizocilpine; NaHS, sodium hydrosulfide; μM, μmol/L.

SH‐SY5Y cells were treated with MK801 for inducing a cell model of SZ, and the protective effect of H_2_S against MK801‐induced cell damage was also evaluated. Results from the CCK‐8 assay indicated that viability decreased significantly in cells treated with 100 μmol/L (μM) or 200 μM of MK801 compared to the normal control group (Figure 3A). For subsequent cellular experiments, MK801 at 100 μM was selected, and the cells were pretreated with different concentrations of NaHS (Figure 3B). In the absence of MK801, different concentrations of NaHS treatment did not affect cell viability significantly (Figure 3B). Under 100 μM MK801 treatment, cell viability significantly reduced, and pretreatment with NaHS at 100 μM enhanced cell viability significantly compared with that in the 0 μM NaHS treatment (Figure 3B). Observations of cell morphology using an inverted microscope were consistent with the results of cell‐viability assays (Figure 3C). Cells induced by MK801 transition from a healthy morphology characterized by an epithelial‐like appearance, short protrusions, and a tendency to grow in clusters, to a damaged morphology characterized by reduced or even disappearing protrusions, cell membrane shrinkage, and even cell death. However, pretreatment with NaHS significantly reversed the morphological changes induced by MK801 (Figure 3C).

#### 3.2.2 Transcriptomic Sequencing, Combined With Bioinformatics Analysis, Reveals H_2_S‐Regulated Genes Enriched in Apoptotic Pathways in SH‐SY5Y Cells

A transcriptomic sequencing approach was employed to assess the expression of mRNA and miRNA across the various groups in SH‐SY5Y cells. A differential expression analysis was conducted on mRNAs between the MK801 group and the control group, between the NaHS+MK801 group and the MK801 group (Figure S1A–E), as well as differentially expressed miRNAs (Figure S1F–J) and the target mRNAs of differentially expressed miRNAs (Figure S1K–L). This strategy led to the identification of 68 key differentially expressed genes (DEGs) regulated by H_2_S (Figure S1M). Analysis of the functional enrichment of DEGs indicated that, in terms of biological processes, they were primarily associated with “transcriptional regulation,” “apoptotic processes,” “cell cycle,” and “neural system development.” In terms of cellular components, they were mainly concentrated in the “cell nucleus” and “cell membrane.” In terms of molecular functions, they were chiefly related to “protein binding” (Figure 1N). Analysis of signaling‐pathway enrichment using the KEGG (Kyoto Encyclopedia of Genes and Genomes) database suggested that, among the top‐20 significantly enriched signaling pathways, “apoptosis” was the most prominently enriched, along with three pathways related to apoptosis (Figure 1O).

#### 3.2.3 H_2_S Inhibits the Increase of Apoptosis Induced by MK801 in SH‐SY5Y Cells

Flow cytometry was employed to assess the impact of H_2_S and MK801 on apoptosis levels in SH‐SY5Y cells. Compared to the control group, the apoptosis level was significantly elevated in the MK801 group, while it was reduced in the NaHS+MK801 group relative to the MK801 group. The administration of NaHS alone had no effect on apoptosis (Figure 3D,E).

### 3.3 Animal Study

#### 3.3.1 H_2_S Alleviates Behavioral Abnormalities in a Rat Model of SZ

**FIGURE 4:**
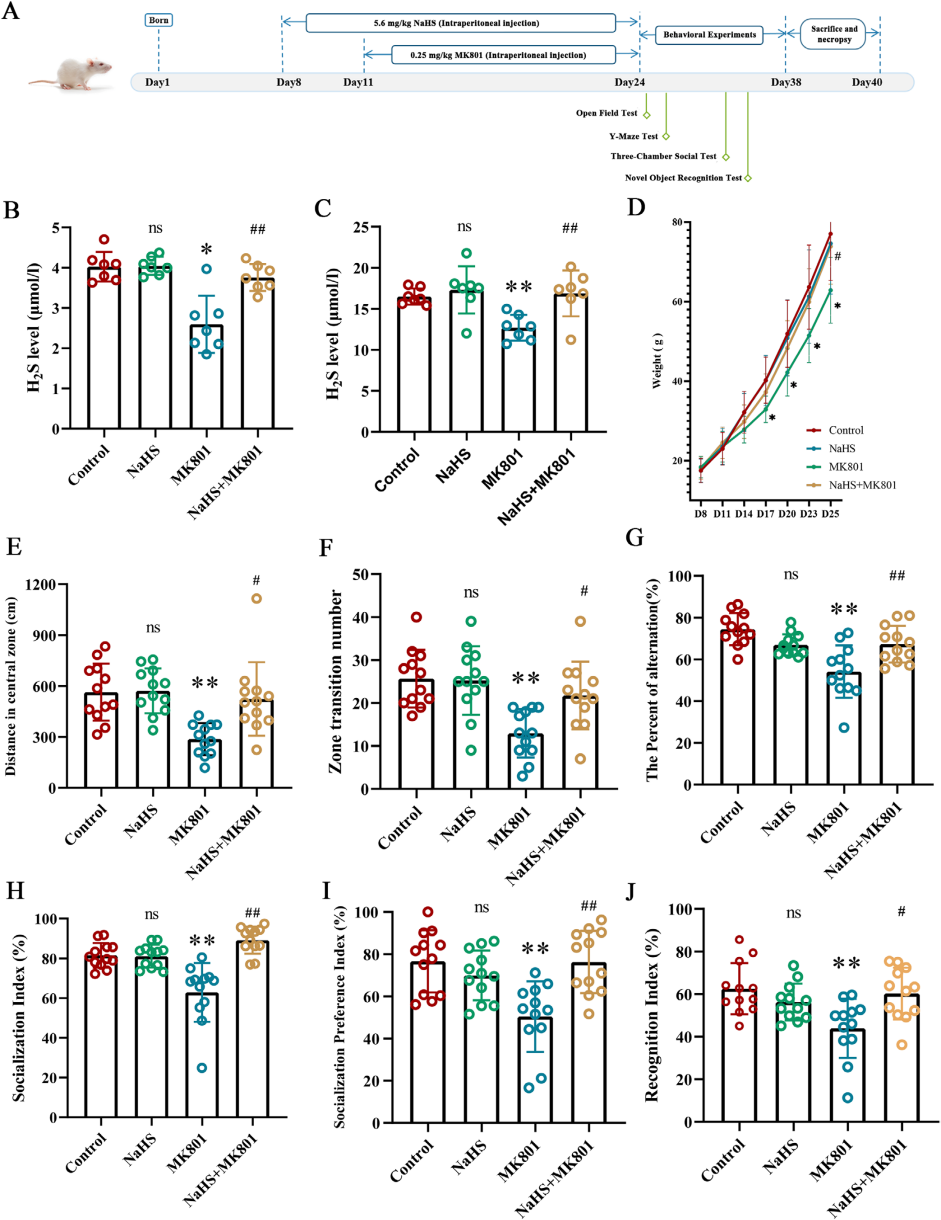
The effect of H_2_S on SZ model rat caused by MK801. (A) Time flow chart of animal experiments. (B) H_2_S content in plasma of rat. *N* = 7. One‐way ANOVA followed by Least Significant Difference(LSD) post hoc analysis, *p < 0.05, ns (no significance) vs. Control, ^##^ *p* < 0.01 vs. MK801. (C) H_2_S content in hippocampus. *N* = 7. One‐way ANOVA followed by Least Significant Difference(LSD) post hoc analysis, **p < 0.01, ns (no significance) vs. Control, ^##^ *p* < 0.01 vs. MK801. (D) Changes in body weight of rats. *N* = 12. One‐way ANOVA followed by Least Significant Difference(LSD) post hoc analysis, *p < 0.05, **p < 0.01, ns (no significance) vs. Control; ^#^ *p* < 0.05 vs. MK801. (E, F) Open field test. (E) Distance in central zone. *N* = 12. Mann–Whitney U test, **P < 0.01, ns (no significance) vs. Control, ^#^ *p* < 0.05 vs. MK801. (F) Zone transition number. *N* = 12. One‐way ANOVA followed by Least Significant Difference(LSD) post hoc analysis, ***P < 0.001, ns (no significance) vs. Control, ^##^ *p* < 0.01 vs. MK801. (G) Y‐maze test. *N* =12. One‐way ANOVA followed by Least Significant Difference(LSD) post hoc analysis, ***p < 0.0001, ns (no significance) vs. Control, ^##^ *p* < 0.01 vs. MK801. (H,I) Three boxes of social experiments. (H) Socialization Index. *N* = 12. Mann–Whitney U test, **p < 0.01, ns (no significance) vs. Control, ^###^ *p* < 0.001 vs. MK801. (I) Social preference index. *N* = 12. One‐way ANOVA followed by Least Significant Difference(LSD) post hoc analysis, ***P < 0.001, ns (no significance) vs. Control, ^###^ *p* < 0.001 vs. MK801. (J) New object recognition experiment. *N* = 12. One‐way ANOVA followed by Least Significant Difference(LSD) post hoc analysis, ***p < 0.001, ns (no significance) vs. Control, ^##^ *p* < 0.01 vs. MK801. ANOVA, Analysis of Variance; MK801, Dizocilpine; NaHS, sodium hydrosulfide.

Wished to further investigate the mitigating effects of H_2_S on SZ and its underlying mechanisms, we constructed a rat model of SZ using MK801 and initiated NaHS treatment 3 days previously. Rats began receiving NaHS (5.6 mg/kg) 8 days after birth, followed by MK801 treatment from day 11 (to construct the SZ model) and continued until day 24. Behavioral experiments commenced thereafter, culminating on day 38 with the sacrifice and necropsy of rats and collection of brain tissue for subsequent experiments (Figure 4A). Initially, we examined the change in the H_2_S level in the blood and hippocampal tissue of rats. We noted a significant reduction in endogenous H_2_S content in the plasma and hippocampus of the MK801 group compared with that in the control group. NaHS pretreatment reversed the MK801‐induced reduction in the H_2_S level in plasma and the hippocampus. Administration of NaHS alone did not affect the H_2_S level in the peripheral blood or hippocampus of rats (Figure 4B,C). Since postnatal day 7 (PND7) onwards, the body weight of rats in the MK801 group exhibited a significant decrease compared to the control group. By PND25, the NaHS+MK801 group demonstrated a reversal of the attenuated weight gain induced by MK801. However, no notable differences were observed between the NaHS and control groups. These findings indicate that MK801 retards weight gain in young rats, while exogenous NaHS supplementation mitigates this effect. Behavioral experimental results suggested that MK801 could induce a reduction in the spontaneous activity in rats (OFT) (Figure 4E,F), diminish exploratory ability in novel environments, and reduce social interest. Social memory (TCST) (Figure 4H,I), spatial working memory (YMT) (Figure 4G), and new object recognition memory (NORT) (Figure 4J) were also impaired. Pretreatment with NaHS could alleviate the aforementioned behavioral changes induced by MK801.

#### 3.3.2 H_2_S Inhibits the Increase of Apoptosis Levels in the Hippocampal Region Induced by MK801 in Rats

**FIGURE 5:**
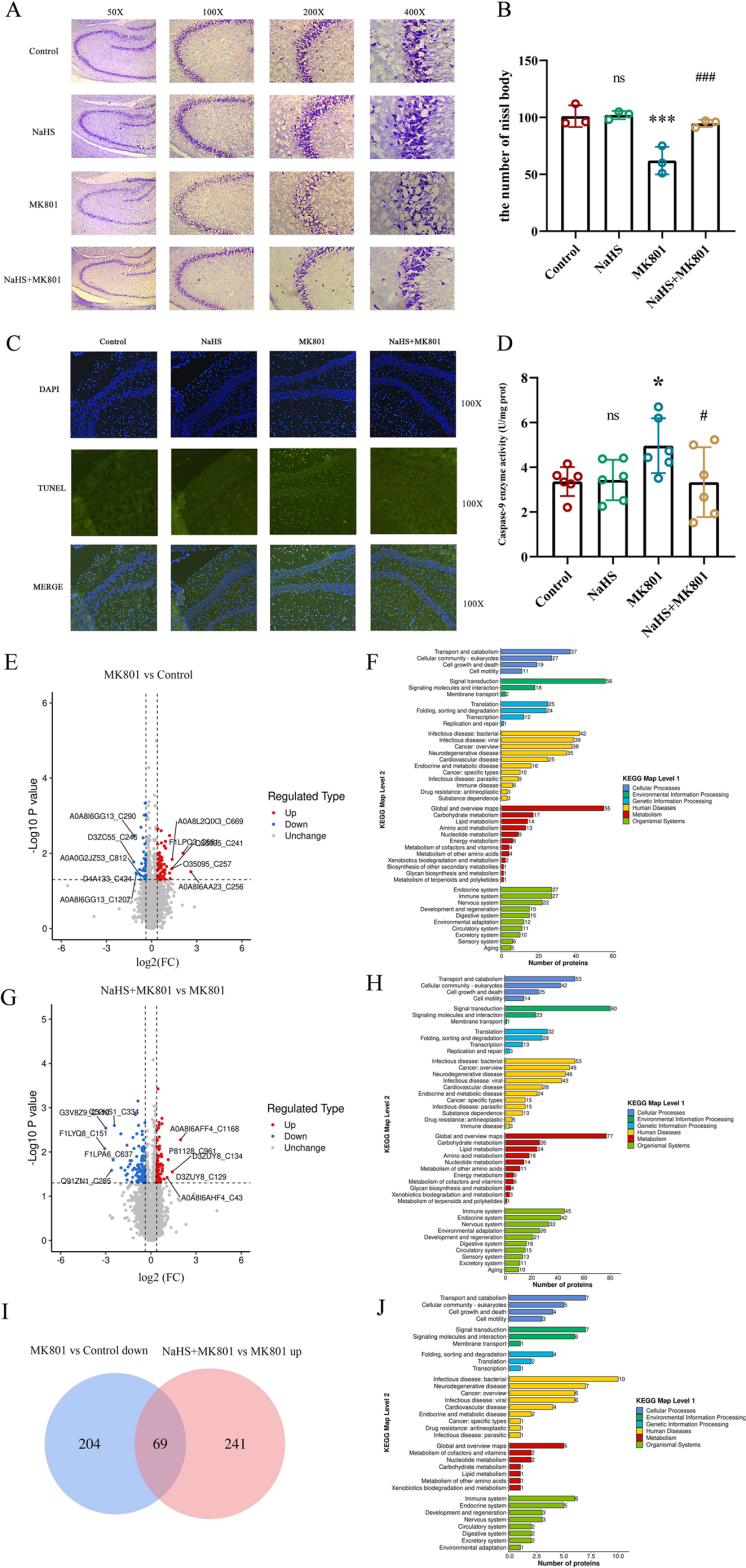
NaHS alleviated apoptosis induced by MK801 and the effect of H_2_S on the S‐sulfhydrylation of proteins in the hippocampal tissue of the rat model of SZ. (A) Nissl staining of rat brain hippocampal. (B) Assessment of the number of Nissl bodies. *N* = 3. One‐way ANOVA followed by Least Significant Difference(LSD) post hoc analysis, ***P < 0.001, ns (no significance) vs. Control, ^##^ *p* < 0.01 vs. MK801. (C) TUNEL staining of rat brain hippocampal. (D) Detection of enzyme activity of apoptosis protein Caspase‐9. *N* = 6. One‐way ANOVA followed by Least Significant Difference(LSD) post hoc analysis, *p < 0.05, ns (no significance) vs. Control, ^#^ *p* < 0.05 vs. MK801. (E) Volcanic maps of different S‐sulfhydrylation modification sites between the MK801 group and the Control group. (F) KEGG pathway analysis of differentially S‐sulfhydrylation modified proteins in the MK801 group and the Control group. (G) Volcanic maps of different S‐sulfhydrylation modification sites between the NaHS+MK801 group and the MK801 group. (H) KEGG pathway analysis of differentially S‐sulfhydrylation modified proteins in the NaHS+MK801 group and the MK801 group. (I) Venn diagram comparing down‐regulated S‐sulfhydrylation modification sites in MK801 vs. Control with up‐regulated S‐sulfhydrylation modification sites in NaHS+MK801 vs. MK801. (J) KEGG pathway analysis of 69 overlapping S‐sulfhydrylation modified proteins in Figure E. ANOVA, Analysis of Variance; DAPI, 2‐(4‐Amidinophenyl)‐6‐indolecarbamidine dihydrochloride; TUNEL, Terminal dUTP Nick End Labeling; KEGG, Kyoto Encyclopedia of Genes and Genomes; MK801, Dizocilpine; NaHS, sodium hydrosulfide.

Subsequently, we utilized Nissl staining to evaluate neuronal damage. We discovered that hippocampal neurons in the control group exhibited a distinct profile, a dense configuration, and a considerable quantity of Nissl bodies. In contrast, the hippocampal neurons in the MK801 group presented unclear contours, a sparse and disordered distribution, and a significantly decreased number of Nissl bodies. The pretreatment group with NaHS effectively mitigated the neuronal damage caused by MK801. These findings imply that NaHS pretreatment can mitigate hippocampal neuron damage in rats with SZ (Figure 5A,B). TUNEL staining was used to detect apoptosis in hippocampal tissue. Treatment with MK801 induced an increase in TUNEL staining in rat hippocampal tissue, indicating elevated levels of apoptosis. Pretreatment with NaHS reduced the level of TUNEL staining, suggesting that H_2_S can mitigate apoptosis caused by MK801 (Figure 5C). The results of the apoptotic protein caspase‐9 activity assay indicated a significant increase in caspase‐9 activity in hippocampal tissues from MK801‐treated rats compared to healthy control rats, suggesting increased apoptosis. The same result showed that pretreatment with NaHS decreased the activity of caspase‐9, suggesting that H_2_S could alleviate the apoptosis induced by MK801 (Figure 5D).

#### 3.3.3 Effect of H_2_S on Protein S‐Sulfhydrylation From Hippocampal Tissue in SZ Model Rats

To further explore the regulatory mechanism whereby H_2_S alleviates apoptosis in SZ, we examined the differential expression of sulfhydryl sites in the hippocampal tissue of rats treated with MK801 and in the control group, as well as the NaHS+MK801 group and MK801 group (Figure 5E,G). Analysis of signaling‐pathway enrichment using the KEGG database revealed that proteins with different modifications were enriched in cellular processes such as “transport and metabolism,” “cell communication,” and “cell growth and death” (Figure 5F,H). There were 69 sites that down regulated in MK801 vs. control and up‐regulated in NaHS+MK801 vs. MK801 (Figure 5I), and they were enriched in “transport and metabolism,” “cell communication,” and “cell growth and death” (Figure 5J). Among the pathways of cell growth and death, four proteins were identified: S‐phase kinase associated protein 1 (Skp1), equestosome 1 (Sqstm1), transferrin (Tf), and Baculoviral IAP Repeat Containing 6 (BIRC6). The latter was involved in the apoptotic pathway (Table S1).

## 4 Discussion

In this study, we delved into the modulatory effects and mechanisms of H_2_S on SZ across human, cellular, and animal models. We discovered a marked and abnormal decrease in the H_2_S level in SZ patients and SZ model rats. Exogenous H_2_S supplementation could alleviate behavioral anomalies and cognitive impairments in rats with SZ. Furthermore, H_2_S reduced the increased apoptosis triggered by MK801 in cell cultures and rat hippocampal tissue. H_2_S augmented the S‐sulfhydrylation of the apoptosis inhibitor protein BIRC6. Collectively, our findings suggest that H_2_S can reduce SZ‐like behavior by modulating apoptosis via S‐sulfhydrylation modifications.

Endogenous H_2_S serves as a vital gasotransmitter with significant physiologic functions. Under pathologic conditions, the dynamic equilibrium of H_2_S is disrupted, leading to alterations in its level within the body. The level of H_2_S decreases in the peripheral blood of patients with depression [29], and H_2_S production decreases in the hippocampal brain tissue of rats subjected to the chronic unpredictable mild stress model of depression [30]. Xiong et al. found that the plasma H_2_S level was reduced significantly in patients with SZ [31]. The H_2_S levels were significantly negatively correlated with the total PANSS score and positively correlated with working memory, visual memory, and executive function [17]. We used unmedicated SZ patients to confirm that the difference in the plasma H_2_S level between HCs and patients with SZ could be attributed solely to the disease itself, thereby excluding drug‐induced effects. Future studies can include a comparison of the H_2_S level in patients after treatment and recovery to investigate if the H_2_S level is related to recovery from disease and the possible impact of treatment on the H_2_S level. In our SZ model rats, we observed a reduction in the H_2_S level in peripheral blood and hippocampal tissue (Figure 4B,C), consistent with the trend in the peripheral blood of patients with SZ, suggesting that SZ disease leads to a decrease in the H_2_S level. However, Ide et al. reported that rats overexpressing mercaptopyruvate sulfurtransferase (MST) exhibited schizophrenia‐like behaviors [32]. They also found an upregulation in the H_2_S production system in brain‐tissue samples from individuals with SZ [32]. However, the H_2_S level in the brain was not measured due to the limitation of sample and measurement methods [32]. Further research may be necessary to confirm the changes in H_2_S levels in the brains of patients with SZ. We hypothesize that an abnormal H_2_S concentration (high or low) can trigger disease phenotypes.

Exogenous administration of H_2_S has a mitigating effect on various diseases. In animal models of AD, the H_2_S level is reduced, and exogenous supplementation of H_2_S can improve cognitive impairment [33]. We found that H_2_S pretreatment could mitigate MK801‐induced SZ‐like behaviors in rats (Figure 4E–J), indicating that H_2_S had a mitigating effect on SZ symptoms. Moreover, NaHS pretreatment could reverse the reduction in H_2_S content in the plasma and hippocampal region of SZ model‐group rats (Figure 4B,C), further confirming that the mitigating effect of H_2_S on SZ symptoms was due to a change in the endogenous H_2_S level.

As a psychiatric syndrome, SZ patients exhibit abnormal apoptotic activity in brain tissue [34]. Neuronal apoptosis is a common phenotype of SZ, and susceptibility and expression levels of apoptosis‐related genes have been reported at the genetic and molecular levels in SZ [35]. Elevated levels of apoptosis have been detected in the cortical cells and serum of SZ patients [36, 37]. Our use of MK801 to treat SY5Y cells resulted in reduced cell viability (Figure 3A–C) and apoptotic phenotypes (Figure 3D,E). Treatment of rats with MK801 resulted in increased apoptosis levels and the activity of apoptosis protein caspase‐9 in the hippocampus (Figure 5C,D), indicating that there is indeed an abnormal increase in apoptosis levels in SZ. The apoptosis levels and the neuronal damage of rats pretreated with NaHS were significantly decreased (Figure 5A,B).

H_2_S can regulate various psychiatric and neurological disorders by inhibiting the apoptotic process. H_2_S has been shown to inhibit cell apoptosis and promote neuronal survival in multiple diseases [38]. It can suppress the expression of the pro‐apoptotic protein caspase‐3 in damaged neurons, playing a neuroprotective role in traumatic brain injury (TBI) [39]. H_2_S reduces neuronal apoptosis in spinal cord injury by activating the nuclear factor erythroid 2‐related factor 2‐dependent signaling pathway [40]. It can prevent neurodegeneration in a mouse model of PD through its antioxidant and antiapoptotic properties [18]. We found that H_2_S could alleviate the reduction in cell viability and the increase of cell apoptosis induced by MK801 (Figure 3), and the H_2_S‐regulated target genes are enriched in the apoptotic pathway (Figure S1N–O). In rats, H_2_S inhibits the increase of apoptosis levels in the hippocampal region induced by MK801 (Figure 5C,D) and reverses SZ‐like behaviors (Figure 4G), indicating that H_2_S plays a mitigating role in SZ by regulating apoptosis.

S‐sulfhydrylation modification regulates the growth, differentiation, apoptosis, and other biological processes involved in signal transduction and metabolic pathways of cells. Therefore, S‐sulfhydrylation modification has a role in the onset and progression of diseases. S‐sulfhydrylation modification of glycogen synthase kinase 3 beta by H_2_S exerts a neuroprotective effect in AD [41]. H_2_S regulates PD through the S‐sulfhydrylation modification of Parkin protein [42]. H_2_S exerts an antiapoptotic effect by promoting the binding of an antiapoptotic transcription factor subunit (nuclear factor kappa B) with the coactivator ribosomal protein S3 through S‐sulfhydrylation modification [43]. We found changes in the S‐sulfhydrylation level of apoptosis‐related proteins. Expression of the apoptosis‐related protein BIRC6 was downregulated in the hippocampal tissue of the SZ model group of rats. S‐sulfhydrylation modification of BIRC6 was upregulated after NaHS pretreatment in the SZ model group (Table S1). BIRC6 is an antiapoptotic protein that can inhibit apoptosis by degrading apoptotic proteins. BIRC6 is expressed widely in oligodendrocytes, inhibitory neurons, and excitatory neurons, and can promote the survival of hippocampal neurons [44]. BIRC6 is associated with various psychiatric and neurological disorders, such as Autism, AD [45], and cognitive functions. For example, BIRC6 affects the development of malnourished axons in AD by regulating the fusion of autophagosomes and lysosomes, thereby affecting cognitive abilities [44]. Our study results indicate that H_2_S exerts a mitigating effect on SZ by regulating apoptosis through the S‐sulfhydrylation modification of BIRC6.

According to the glutamate hypothesis of SZ, alterations of the NMDA receptor in inhibitory GABAergic interneurons can cause dysfunction of GABAergic interneurons and lead to disinhibition of excitatory neurons. On the one hand, the disinhibition of dopaminergic neurons in the striatum could cause positive symptoms. On the other hand, the disinhibition of glutamatergic neurons in the cerebral cortex could produce a significant release of glutamate and trigger an increase in apoptosis signals subsequently, resulting in abnormal synaptic pruning, which may be the cause of negative symptoms and cognitive impairment in SZ [46]. Therefore, the abnormality of NMDAR is an important pathological basis of SZ. MK801, an antagonist of NMDAR, can be used to simulate SZ in rats. The effect of H_2_S on NMDAR has been reported. H_2_S enhanced the NMDAR‐mediated responses and induced the hippocampal long‐term potentiation (LTP) [47]. In addition, H_2_S and MK801 had the same ameliorative effect on homocysteine (Hcy)‐induced cognitive impairment and could inhibit the Hcy‐induced increase of NMDAR1 gene and protein level [48, 49]. The effect of H2S on NMDAR in SZ has not been reported. In this study, we focus on the mechanism of H_2_S alleviating SZ through apoptotic signaling; however, the alteration of NMDAR in SZ is closely related to apoptosis signals, and whether H_2_S affects apoptosis signaling by regulating the activity of NMDAR is worthy of further study.

## 5 Conclusion

In conclusion, we employed samples from humans, cells, and animals to investigate the role of H_2_S in SZ. H_2_S exerted a regulatory effect on SZ by inhibiting apoptosis through S‐sulfhydrylation modification. However, our research had two main limitations. First, we examined the change in the H_2_S level in the peripheral blood and hippocampal brain tissue of the study population and animal models. Further research is needed to confirm whether the H_2_S level in human peripheral blood is consistent with changes in the central nervous system. Second, H_2_S has shown potential therapeutic effects in various diseases. However, due to its volatility and oxidization ability, the stability of H_2_S in disease treatment (as well as issues related to the dosage, method of administration, and safety of exogenous administration) requires further exploration to develop a stable and standardized system. H_2_S demonstrated important therapeutic potential in animal experiments, but its clinical efficacy remains to be proven.

## Author Contributions

Conceptualization‐Sha Liu and Yong Xu; writing‐original draft preparation‐Xinzhe Du, Wei Hu, Meiqi Liu, and Jinzhi Lv; writing‐review and editing‐Xinzhe Du and Sha Liu; supervision‐Yao Gao, Xiao Wang, Wentao Zhao, Junxia Li, Xinrong Li, Xiaohua Cao, Zhifen Liu, Yong Xu, and Sha Liu.

## Ethics Statement

This study was approved by the Research Ethics Committee of the First Hospital of Shanxi Medical University in accordance with the Declaration of Helsinki, and consent to participate was obtained from all participants. Patients with SZ and HC were invited to take part in the study provided that they met the enrollment criteria. There was no charge for participation, and all subjects signed the informed consent.

## Conflicts of Interest

The authors declare no conflicts of interest.

## Supporting information


Data S1.

## Data Availability

The data that support the findings of this study are available from the corresponding author upon reasonable request.
