# Supplementary material for: Hydrogen Sulfide Alleviates Schizophrenia‐Like Behavior Through Regulating Apoptosis by S‐Sulfhydrylation Modification
Source: CNS Neurosci Ther. 2025 Feb 18;31(2):e70278. doi: 10.1111/cns.70278 (PMC11833453; doi:10.1111/cns.70278)
Supplement: Supplementary file 1 — Data S1. [file CNS-31-e70278-s001.docx]

**Materials and methods**

**Human subjects**

**Participants**

The study included three population cohorts with a total of 78 patients with schizophrenia (SZ) and 83 healthy controls (HC), including two initial discovery cohorts and a late validation cohort. The discovery cohort refers to the initial group of subjects or samples used in research to discover new findings. The patients were recruited from First Hospital of Shanxi Medical University, and the HC were recruited from the community according to the following criteria.

The inclusion and exclusion criteria for SZ:

The inclusion criteria were: (1) Diagnosed as having SZ by at least two experienced psychiatrists according to the Chinese version of the fifth edition of the Diagnostic and Statistical Manual of Mental Disorders (DSM-5)^1^; (2) The first episode of SZ and no history of drug treatment; (3) Aged 13–60 years; (4) Positive and Negative Syndrome Scale (PANSS) score ≥60 points; (5) The Han nationality; (6) Right-handed.

The exclusion criteria were: (1) Patients with other mental disorders and neurological disease; (2) Patients with organic brain disease including epilepsy, dementia, brain trauma, brain tumour and encephalitis; (3) Substance dependence or abuse such as liquor, nicotine, psychoactive drugs, etc.; (4) Diseases of the endocrine system, the blood system, the kidney system, the immune system, and other serious physical diseases; (5) Women who are pregnant or breastfeeding.

The inclusion and exclusion criteria for HC:

The inclusion criteria were: (1) Individuals did not meet the clinical diagnostic criteria for schizophrenia according to DSM-V; (2) Aged 13–60 years; (3) The Han nationality; (4) Right-handed.

The exclusion criteria were: (1) Individuals with history or current presence of mental illness; (2) Individuals with family history of mental illness; (3) Substance dependence or abuse such as liquor, nicotine, psychoactive drugs, etc.; (4) Individuals with organic brain disease including epilepsy, dementia, brain trauma, brain tumour and encephalitis; (5) Individuals with serious unstable physical illnesses, diagnosed diabetes, thyroid disease, hypertension, heart disease, and so on.

Participants underwent physical examinations and provided demographic data including age, sex, education, history of mental disorders, body mass index (BMI), blood pressure (BP), prevalence of comorbidity, substance dependence or abuse and blood samples. SZ patients also provided information on whether they had other comorbidities. Psychiatric symptoms of SZ patients were evaluated using the PANSS (Positive and Negative Syndrome Scale). Ethical approval of the study protocol was granted by the Ethics Committee of First Hospital of Shanxi Medical University (ChiCTR900026776). Informed consent obtained from all participants.

**Methylene blue method**

The content of hydrogen sulfide (H_2_S) in 100μL of human plasma was quantified using the methylene blue method as described previously^2^.

**Enzyme Linked Immunosorbent Assay (ELISA)**

The concentration of H_2_S in human peripheral blood was determined using a human H_2_S ELISA kit (Wuhan Xinqidi Biotech Co.LTD, Wuhan, China). Standard samples and specimens were placed in an antibody-coated 96-well plate and incubated at 37°C for 90 min. Following this action, each well received 60-min incubation with biotinylated H_2_S antibody solution. After washing, plates underwent 30-min incubation with horseradish peroxidase-bound avidin. Color development was initiated with tetramethylbenzidine and halted using a termination solution. Absorbance at 450 nm at 37°C was measured to determine the H_2_S concentration by correlation with the absorbance value using a standard curve.

**Cell studies**

**Culture and maintenance of cells**

We utilized SH-SY5Y cells from the Shanghai Cell Bank of the Chinese Academy of Sciences (Beijing, China). SH-SY5Y cells were cultured in RPMI 1640 medium with 15% fetal bovine serum. At >95% confluence, trypsinization and passage at a 1:3 ratio was undertaken, with subsequent incubation at 37°C in an atmosphere of 5% CO_2_.

**Cell Counting Kit (CCK)8 assay**

The CCK-8 assay was employed to measure toxicity to SH-SY5Y cells. We established the MK801 concentration which caused notable cell damage (~70% cell viability). We also determined the NaHS concentration that reduced MK801-induced cell damage. 100 μL cell suspension (about 5000 cells)/well was added into the 96-well plate, incubated for 24 hours, and 10 μL/ well CCK-8 solution was added, incubated for 2.5 hours, and absorbance was measured at 450nm.

**High-throughput sequencing of miRNA and mRNA**

Sangon Biotech (Shanghai) Co., Ltd. conducted quality control, library construction, and transcript sequencing. Sequencing was undertaken on the Hiseq™ platform (Illumina, San Diego, California, United States of America (USA)). Sequencing was followed by hierarchical indexing for spliced alignment of transcripts (HISAT) for aligning to the human reference genome, transcript retrieval, and quantification of gene expression. Differential gene expression was analyzed using Differential Expression Sequencing (DESeq) with Benjamini–Hochberg-adjusted P-values. The Targetscan database ([www.targetscan.org/](http://www.targetscan.org/)) was employed for prediction of miRNA targets. The Gene Ontology (GO) database (<https://geneontology.org/>) and Kyoto Encyclopedia of Genes and Genomes (KEGG) database ([www.genome.jp/kegg/](http://www.genome.jp/kegg/)) were used to identify enrichment of function and signaling pathways, respectively, *via* Unikawa Biocloud (<http://www.omicstudio.cn/doc/1152>).

**Apoptosis test**

Cells were stained using the Annexin V conjugated with Fluorescein Isothiocyanate (Annexin V-FITC) and Propidium Iodide (PI) Apoptosis Detection Kit (Yeasen Biotechnology (Shanghai) Co., LTD). Briefly, after digestion, washing with cold phosphate-buffered saline, and suspension in binding buffer, Annexin V-FITC and PI staining solution was added. When the reaction had ended, binding buffer was added, cells placed on ice, and analyzed by flow cytometry within 1 hour.

**Animal experiments**

**NaHS treatment**

The study protocol was approved (2021-150) by the Animal Ethics Committee of Shanxi Medical University (Shanxi, China). In the Specific Pathogen-Free Animal Center of the Mental Health Laboratory of Shanxi Medical University First Hospital, animals received optimal care in a room at a controlled temperature of 24°C and relative humidity of 55%. Twelve Sprague–Dawley rats were acquired from SPF (Beijing) BIOTECHNOLOGY Co., Ltd. *Postpartum*, male offspring were categorized into four groups of 12: control (received physiologic (0.9%) saline (2 mL/kg bodyweight, postnatal day (PND)8–24)); NaHS (5.6 mg/kg, PND8–24), MK801 (0.25 mg/kg, PND11–24), and NaHS+MK801 (injection of NaHS and MK801 at an interval of 1 h). Rats received agents by intraperitoneal injection.

**Behavioral tests**

Video tracking of the animals (SMART 3.0 system, Panlab, Spain, a subsidiary of Harvard apparatus, United States of America (USA)) was carried out to analyze behavioral results.

**Open Field Test (OFT)**

A behavioral box segmented into peripheral and central zones, served as the arena in which rats roamed uninhibited for 5 min. The duration and frequency of activity in the central zone were documented to evaluate autonomous movement, environmental exploration, stress, anxiety, and depression-like behavior.

**Y-Maze Test (YMT)**

The apparatus was employed to assess spatial memory and working memory in rats. Comprising three uniform arms, the maze centered on a triangular space in which rats were placed and permitted an 8-min exploration. The spontaneous alternation accuracy rate was determined by the formula:

spontaneous alternation accuracy rate = [number of correct alternations/(total number of entry arms − 2)] × 100%

**Three-Chamber Social Test (TCST)**

The study comprised three sequential 10-min phases. Initially, rats were allowed to acclimatize in identical cages at opposite ends of a box. The second phase involved timing the exploration of an unfamiliar rat (R1) and an empty cage. A new rat (R2) was introduced in the third phase, and interactions between R1 and R2 were timed. The Social Index was calculated as the percentage of time with R1 over total exploration time. The Social Preference Index reflected the proportion of time with R2 over all R1 and R2 interaction times (R2/(R1+R2)).

**Novel Object Recognition Test (NORT)**

The experiment evaluated cognitive memory in a chamber of dimension 60 cm × 60 cm × 50 cm. Initially, rats explored two identical objects for 10 min. After six hours, one object was swapped with a novel one, and the short-term recognition and memory were gauged over 10 min. This study was conducted to assess the cognitive memory of rats. The NORT was calculated as:

NORT = Time spent exploring new objects/(time spent exploring new objects + time spent exploring old objects) ×100%

**Fluorescence staining**

Apoptosis was assessed using the Terminal deoxynucleotidyl transferase deoxyUridine Triphosphate (dUTP) Nick End Labelin (TUNEL) assay. Hippocampal sections from rats (thickness = 10 μm) were fixed in 4% paraformaldehyde and then stained with the TUNEL kit (Alexa Fluor 488; Yeasen Biotechnology, Shanghai, China). After staining, sections were treated with a 4′,6-diamidino-2-phenylindole-containing anti-fluorescence quencher for nuclear visualization and examined under a fluorescence microscope (Leica, DM2000, Wetzlar, Germany).

**Nissl staining**

Sections (10μm thick) were prepared. The sections were fixed in 4% paraformaldehyde and stained with 10% Cresyl violet for 50 minutes at 56°C. Subsequently, the sections were dehydrated using alcohol with varying concentration gradients and finally sealed. The images were captured using an upright microscope.

**Caspase-9 activity**

A caspase-9 activity analysis kit (colorimetric) is based on the ability of caspase-9 to catalyze the substrate acetyl-Asp-Glu-Val-Asp-p-nitroanilide (Ac-LEDH-pNA). This catalysis results in the production of yellow *p*-nitroaniline (pNA). pNA strongly absorbs light near 405 nm, which enables the detection of caspase-9 activity.

**S-sulfhydrylation detection**

Jingjie PTM Biolab (Hangzhou) Co. Inc conducted the detection. Proteins in samples were equally digested and volumes matched to lysates. A 1:1 ratio of chilled acetone was added, then quadrupled after vortex-mixing. The mixture was precipitated at −20°C for 2 h, centrifuged at 4500 × *g* for 5 min, and supernatants removed. This step was repeated 2–3 times. After natural air-drying, triethylammonium bicarbonate (200 mM) was used to dissolve the precipitate with the aid of ultrasound. Proteins, with modified (-S-SH) and non-disulfide (-SH) cysteine residues labeled by iodoacetamide-biotin, underwent overnight trypsin digestion at a 1:50 ratio. Resultant peptides in an immunoprecipitation buffer were incubated with resin at room temperature for 1 h, washed twice, and eluted twice at 30°C for 30 min. Eluents treated with iodoacetamide were vacuum-frozen and desalted, followed by liquid chromatography–mass spectrometry with a NanoElute (Bruker, Billerica, Massachusetts (MA), United States of America (USA)).


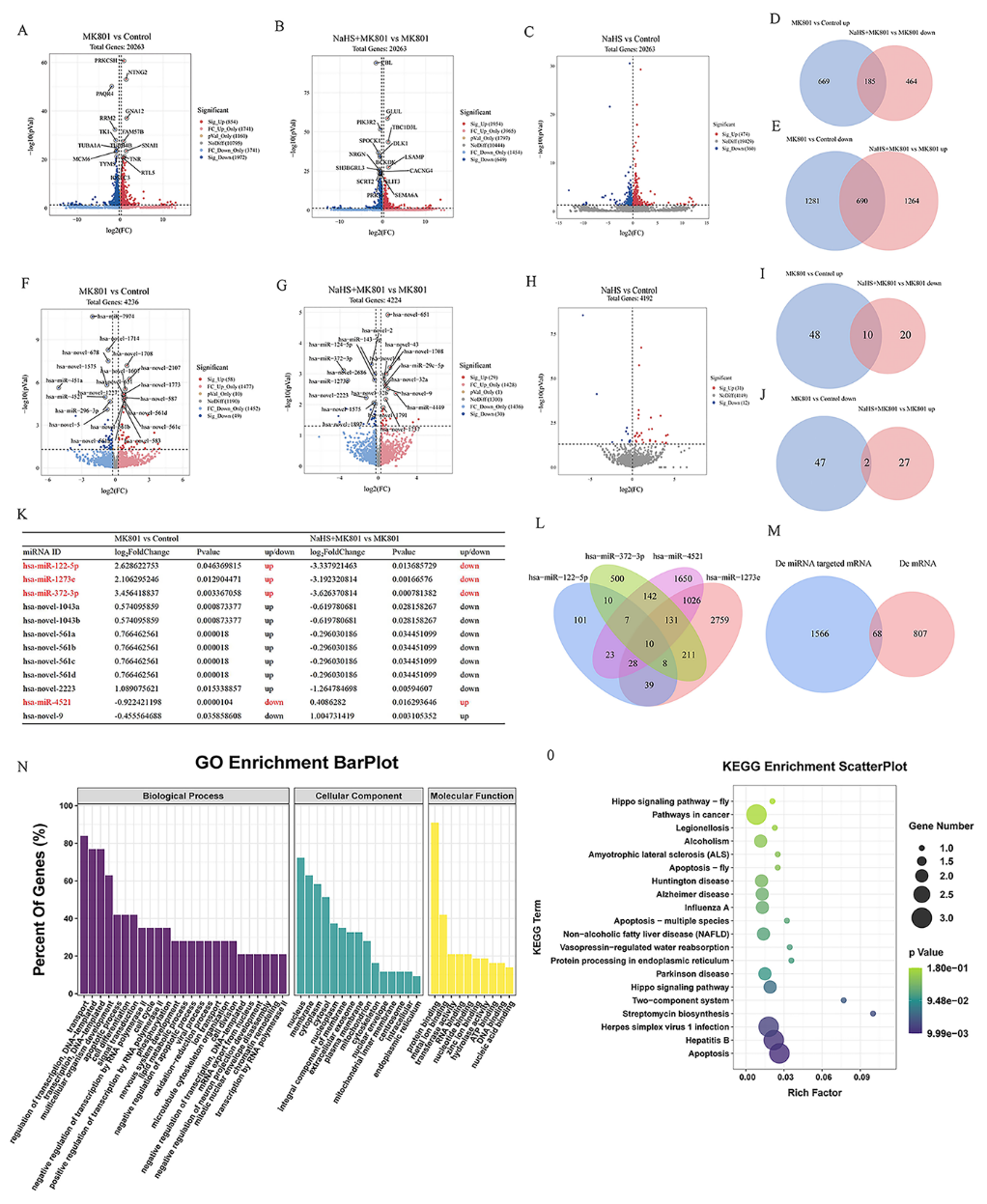


**Supplemental Figure 1. In SY5Y, high-throughput sequencing of miRNA and mRNA revealed the mechanism by which hydrogen sulfide alleviates damage caused by MK801.** A. Volcano plot of DEGs between MK801 treatment and Control group. B. Volcano plot of DEGs between NaHS+MK801 treatment and MK801 treatment. C. Volcano plot of DEGs between NaHS treatment and Control group. D. Venn diagram of overlapping DEGs between up-regulated genes of MK801 vs Control and down-regulated genes of NaHS+MK801 vs MK801. E. Venn diagram of overlapping DEGs between down-regulated genes of MK801 vs Control and up-regulated genes of NaHS+MK801 vs MK801. F. Volcano plot of DE miRNAs beween MK801 treatment and Control group. G. Volcano plot of DE miRNAs between NaHS+MK801 treatment and MK801 treatment. H. Volcano plot of DE miRNAs between NaHS treatment and Control group. I. Venn diagram of overlapping DE miRNAs between up-regulated miRNAs of MK801 vs Control and down-regulated miRNAs of NaHS+MK801 vs MK801. J. Venn diagram of overlapping DE miRNAs between down-regulated miRNAs of MK801 vs Control and up-regulated miRNAs of NaHS+MK801 vs MK801. K. The details of DE miRNAs of overlapping miRNAs in I and J. L. Venn diagram of common target genes of four DE-miRNAs in K. M. Venn diagram of overlapping genes of four DE-miRNAs in K targeted genes and DE-mRNAs from overlapping genes in D and E. N. Go analysis of key mRNAs. O. KEGG pathway analysis of key mRNAs. FC: Fold change. *P* Val: *p* value. Note: NaHS, sodium hydrosulfide; MK801, Dizocilpine; GO, Gene Ontology; KEGG, Kyoto Encyclopedia of Genes and Genomes.

| **KEGG map level** | **Gene name** | **Position** | **MK801/Control Ratio** | **MK801/Control P value** | **NaHS+MK801/MK801**  **Ratio** | **NaHS+MK801/MK801**  **P value** | **KEGG pathway** |
| --- | --- | --- | --- | --- | --- | --- | --- |
| Cell growth and death | Skp1 | 160 | 0.001 | 0.001 | 1000 | 0.0001 | map04110 Cell cycle; map04114 Oocyte meiosis; map04120 Ubiquitin mediated proteolysis; map04141 Protein processing in endoplasmic reticulum; map04310 Wnt signaling pathway; map04341 Hedgehog signaling pathway - fly; map04350 TGF-beta signaling pathway; map04710 Circadian rhythm; map05131 Shigellosis; map05132 Salmonella infection; map05170 Human immunodeficiency virus 1 infection; map05200 Pathways in cancer |
|  | Sqstm1 | 216 | 0.001 | 0.001 | 1000 | 0.0001 | map04137 Mitophagy - animal; map04140 Autophagy - animal; map04217 Necroptosis; map04218 Cellular senescence; map04380 Osteoclast differentiation; map05014 Amyotrophic lateral sclerosis; map05022 Pathways of neurodegeneration - multiple diseases; map05131 Shigellosis; map05418 Fluid shear stress and atherosclerosis |
|  | Tf | 67 | 0.001 | 0.001 | 1000 | 0.0001 | map04066 HIF-1 signaling pathway; map04216 Ferroptosis; map04978 Mineral absorption |
|  | Birc6 | 3920 | 0.001 | 0.001 | 1000 | 0.0001 | map04120 Ubiquitin mediated proteolysis; map04214 Apoptosis - fly; map04215 Apoptosis - multiple species |

Supplemental Table 1. Details of Protein in Cell growth and death pathway.

Note: KEGG, Kyoto Encyclopedia of Genes and Genomes; NaHS, sodium hydrosulfide; MK801, Dizocilpine; Skp1, S-Phase Kinase Associated Protein 1; Sqstm1, Sequestosome 1; TF, Tissue factor; BIRC6, Baculoviral IAP Repeat Containing 6

References:

1. Biedermann F, Fleischhacker WW. Psychotic disorders in DSM-5 and ICD-11. *CNS Spectr.* 2016;21(4):349-354.

2. Du X, Jin Z, Liu D, Yang G, Pei Y. Hydrogen sulfide alleviates the cold stress through MPK4 in Arabidopsis thaliana. *Plant Physiol Biochem.* 2017;120:112-119.
